# Exploring the Role of Nocturnal Hypoxemia and Sleep Fragmentation in Memory Decline: Insights From Explainable Machine Learning Models

**DOI:** 10.1111/crj.70188

**Published:** 2026-05-08

**Authors:** Xin Li, Yingying Zhu, Fansu Meng, Kangan Lai, Yuling Liang, Huang Ting, Zhengnan Mai, Liang Li

**Affiliations:** ^1^ The Tenth Clinical Medical College of Guangzhou University of Traditional Chinese Medicine Zhongshan Hospital of Traditional Chinese Medicine Zhongshan China; ^2^ Doctor of Physiotherapy, University of Sydney Sydney Australia

**Keywords:** machine learning, memory decline, micro‐arousal, nocturnal hypoxemia, polysomnography, sleep fragmentation

## Abstract

**Introduction:**

Sleep‐disordered breathing (SDB) is linked to memory decline, but the exact relationship between sleep fragmentation, nocturnal hypoxemia, and cognitive impairment remains unclear.

**Objectives:**

This study aimed to investigate the associations between micro‐arousal burden, nocturnal oxygen desaturation, and memory decline in patients with moderate‐to‐severe OSA.

**Methods:**

Data were retrieved from the clinical and overnight polysomnographic (PSG) records of adult patients evaluated for suspected SDB. The primary clinical endpoint was the presence and severity of memory decline, ascertained via a standardized Subjective Cognitive Decline (SCD) instrument. A multidimensional array of variables was systematically extracted, encompassing baseline demographic characteristics, cardiometabolic comorbidities, and high‐resolution sleep architecture metrics, with a distinct emphasis on stage‐specific micro‐arousal burdens and the morphological profiles of nocturnal oxygen desaturation. Then, independent *t* tests and $x^{2}$ tests were initially utilized to characterize PSG disparities between the memory‐normal and memory‐decline groups. And interpretable machine learning algorithms, utilizing rigorously partitioned training and validation sets, were deployed to predict cognitive trajectories and elucidate the relative prognostic importance of specific sleep‐related parameters.

**Results:**

The final analytical sample comprised 884 participants with complete primary outcome data (memory‐normal: *N* = 408; memory‐decline: *N* = 476). Initial comparative analyses revealed the memory‐decline group was older (50.24 vs. 45.95 years, *p* < 0.001) with a significantly higher prevalence of cardiometabolic comorbidities, including hypertension (47.3% vs. 40.2%, *p* = 0.035) and diabetes (24.4% vs. 8.8%, *p* < 0.001). Polysomnographically, this group exhibited a distinct hypopnea‐predominant phenotype: despite a comparable overall AHI (45.82 vs. 48.64 events/h, *p* = 0.099) and global arousal index (26.98 vs. 28.85 events/h, *p* = 0.172), they demonstrated a significantly higher hypopnea count (122.25 vs. 110.40, *p* = 0.047) and prolonged awake time with SpO_2_ < 95% (33.71 vs. 27.71 min, *p* = 0.015). Paradoxically, their nadir SpO_2_ was elevated (76.68% vs. 74.39%, *p* = 0.009), maximal obstructive events were shorter (51.42 s vs. 57.49 s, *p* < 0.001), and obstructive desaturation events were fewer (180.33 vs. 219.70, *p* = 0.006), indicating a shift toward shallower, persistent desaturation morphologies. Furthermore, interpretable machine learning models, rigorously evaluated on the independent validation set, identified spontaneous NREM micro‐arousals, total REM micro‐arousals, and obstructive desaturation metrics as the highest‐ranking predictive determinants of memory decline.

**Conclusions:**

Memory decline in SDB is more robustly associated with the morphological profile of oxygen exposure rather than absolute event frequencies. A hypopnea‐dominant profile with mild, persistent low oxygen levels offers an associative framework for understanding cognitive decline. Future research and clinical interventions should prioritize hypoxic burden as a key factor in phenotype identification and memory decline treatment.

## Graphical abstract

This graphical abstract illustrates the study workflow from polysomnography (PSG) data collection to explainable machine learning analysis and cognitive outcomes. Left: PSG monitoring captures nocturnal physiological signals including airflow and oxygen saturation. Center: Explainable AI (XRT algorithm) and SHAP analysis identify spontaneous NREM micro‐arousals, REM micro‐arousals, and obstructive desaturation patterns as key predictors. Right: These sleep fragmentation and hypoxemia features associate with subjective memory decline, highlighting that the morphological profile of oxygen exposure (prolonged mild hypoxemia) rather than event frequency drives cognitive vulnerability.

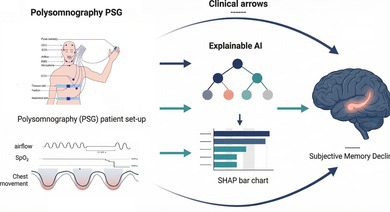

## 1 Introduction

Obstructive sleep apnea (OSA) is a highly prevalent, multifactorial sleep disorder characterized by the interplay between mechanical upper‐airway obstruction, central neurotransmission dysregulation (serotonergic and dopaminergic), and genetic susceptibility, leading to intermittent hypoxemia, sleep fragmentation, and marked reductions in sleep quality [1, 2, 3]. Epidemiological studies indicate that OSA affects a substantial proportion of adults (population prevalence ~9%–38% at AHI ≥ 5), with prevalence escalating in higher‐risk groups such as older individuals and those who are overweight or obese [4, 5]. Beyond well‐established links to cardiovascular and metabolic disease, accumulating evidence shows that OSA is closely associated with a broad spectrum of systemic and molecular perturbations, including neurocognitive impairment—notably deficits in attention, executive function, and memory—as well as electrolyte dysregulation, vitamin D deficiency, elevated uric acid levels, and altered expression of hypoxia‐responsive molecular pathways such as neuronal PAS domain protein 2 (NPAS2) and hypoxia‐inducible factor 1‐alpha [6, 7, 8].

A hallmark neurophysiological consequence of OSA is sleep fragmentation, which, particularly in moderate‐to‐severe cohorts, is predominantly driven by a high burden of micro‐arousals precipitated by respiratory effort and oxygen desaturation events. However, it is increasingly recognized that sleep fragmentation represents a multifactorial phenomenon that can also stem from diverse etiologies beyond respiratory events, including periodic limb movements, neurochemical imbalances, and concurrent primary sleep disorders [9]. Micro‐arousals are brief (3–15 s) transient shifts to lighter sleep (not usually awakening the patient), occurring as physiological responses to disrupted breathing; when excessive in number, these micro‐arousals fragment the normal sleep architecture, producing effects comparable to sleep deprivation (e.g., mood dysregulation and cognitive deficits) [10]. Recent studies have demonstrated that both the burden and the characteristics of micro‐arousals are independently associated with cognitive performance in patients with OSA [11]. For instance, patients with OSA with neurocognitive impairment exhibit significantly greater frequency of longer micro‐arousals (≥ 5–15 s) compared with those without impairment [12]. Moreover, OSA disturbances occurring during specific sleep stages may differentially impact cognition‐for example, OSA‐related arousals and hypoxemia during REM sleep have been linked to worse memory consolidation outcomes than those during NREM sleep [13]. Simultaneously, intermittent nocturnal hypoxemia in OSA is implicated in neurodegenerative changes and poorer cognitive outcomes, likely via oxidative stress, neuronal injury, and vascular dysfunction [14].

Despite these advances, the precise interplay between sleep fragmentation, intermittent hypoxemia, and cognitive impairment in OSA remains incompletely understood. Traditional analyses have focused on isolated severity metrics such as the apnea–hypopnea index (AHI) or average oxygen saturation, which do not fully capture the complex, multidimensional nature of sleep‐disordered breathing and its neurocognitive sequelae [15]. With the advent of interpretable machine learning techniques, it is now possible to integrate a wide array of polysomnographic features to identify key predictors and to provide transparent, data‐driven insights into OSA pathophysiology [16, 17]. Recent studies have utilized explainable ML models (permutation‐based random forests and SHAP analyses) to reveal influential risk factors from sleep data, enabling clinicians to understand which sleep‐related features drive outcomes [18].

In this study, we aimed to systematically investigate the associations between micro‐arousal burden, nocturnal oxygen desaturation, and memory decline in patients with moderate‐to‐severe OSA. We also sought to employ interpretable machine learning models to elucidate the most influential sleep‐related features contributing to micro‐arousal events and cognitive impairment, providing new perspectives for risk stratification and targeted interventions in this vulnerable population.

## 2 Methods

### 2.1 Study Design and Study Population

This retrospective observational study included adult patients who underwent diagnostic overnight polysomnography (PSG) at the Department of Zhongshan Traditional Chinese Medicine Hospital, Guangdong Province, China, from January 2020 to February 2024. Eligible participants were aged 18 years or older, clinically suspected of having sleep‐disordered breathing, and had complete clinical and PSG data available. Exclusion criteria were a primary diagnosis of central sleep apnea, known neurodegenerative or severe psychiatric disorders, current use of sedative or hypnotic medications, or incomplete data. The study was approved by the institutional ethics committee (approval no. 2021ZSZY‐LLK‐131), and all participants provided informed consent.

### 2.2 Definition of Sleep Fragmentation

Sleep fragmentation was quantified utilizing polysomnographic micro‐arousal metrics, in strict accordance with the American Academy of Sleep Medicine (AASM) scoring manual. Micro‐arousals were defined as abrupt, transient shifts in electroencephalographic (EEG) frequency (specifically including alpha, theta, and/or frequencies > 16 Hz, while excluding sleep spindles) lasting for at least 3 s, occurring during both rapid eye movement (REM) and non‐REM (NREM) sleep stages. The total burden of micro‐arousals, alongside stage‐specific counts for REM and NREM sleep, was extracted. Additional parameters, including the arousal index (defined as the number of micro‐arousals per hour of total sleep time), spontaneous arousals, and respiratory event‐related arousals, were initially subjected to automated scoring and subsequently underwent rigorous manual verification and adjudication by certified sleep technologists.

### 2.3 Definition of Nocturnal Hypoxemia and Respiratory Events

Nocturnal hypoxemia was evaluated by several oxygen saturation parameters recorded during PSG. Specifically, hypopneas were scored according to the AASM recommended criteria (Rule A), defined as a peak signal excursion drop by ≥ 30% of pre‐event baseline using nasal pressure lasting for ≥ 10 s, in association with either a ≥ 3% arterial oxygen desaturation or an EEG arousal. Key indices included mean and minimum nocturnal peripheral oxygen saturation (SpO_2_), the NREM oxygen desaturation index (ODI, defined as the number of ≥ 3% oxygen desaturations per hour during NREM sleep), and cumulative time spent with SpO_2_ below 80%, 85%, 90%, and 95%. Hypoxemic episodes associated with apneic or hypopneic events were specifically annotated. All oxygenation metrics followed standardized definitions and cut‐offs per AASM guidelines.

### 2.4 Definition of Memory Decline

Memory decline was evaluated utilizing a standardized self‐administered questionnaire completed concurrently with the PSG assessment. Participants rated changes in their memory compared with baseline using a four‐level ordinal scale: 0 = *no decline*, 1 = *mild decline*, 2 = *moderate decline*, and 3 = *severe decline*. In the clinical context, this patient‐reported outcome measure (PROM) captures the phenotype of Subjective Cognitive Decline (SCD), functioning as an early indicator of neurocognitive vulnerability. For secondary analyses, patients were grouped according to memory decline severity.

### 2.5 Statistical Methods

#### 2.5.1 Baseline Characteristics and Data Preprocessing

Continuous variables are presented as mean ± standard deviation (SD), while categorical variables are summarized as frequencies and percentages. To maximize data utility while mitigating potential imputation bias, missing values were addressed using a stratified imputation strategy based on the primary clinical outcome (memory status). Specifically, missing quantitative variables were imputed using the respective mean of the “Memory‐normal” or “Memory‐decline” group, whereas missing categorical variables were imputed using the group‐specific mode. Participants with missing data on the primary outcome were strictly excluded from the final analytical cohort. Between‐group comparisons of baseline clinical and polysomnographic characteristics were performed using one‐way analysis of variance (ANOVA) for continuous variables and chi‐squared tests for categorical variables, followed by Bonferroni correction for multiple testing. Pearson correlation analysis was employed to evaluate the collinearity and associations among sleep fragmentation, nocturnal hypoxemia, and other relevant polysomnographic indices.

#### 2.5.2 Automated Machine Learning and Model Development

Given the complex, high‐dimensional nature of polysomnographic data, traditional linear models may be insufficient to capture potential non‐linear relationships and clinical thresholds. Therefore, the Automated Machine Learning (AutoML) framework via the h2o package (version 3.46.0.10) was utilized to construct predictive models and identify key predictors of memory decline. The total dataset was randomly partitioned into a training set (65%) for model derivation and an independent internal validation set (35%) for rigorous model evaluation and parameter optimization.

The AutoML pipeline autonomously evaluated diverse model architectures, incorporating regularized Generalized Linear Models (GLM, structurally equivalent to multivariable logistic regression) as the internal baseline benchmark. The optimal model identified was Extremely Randomized Trees (XRT), an advanced variant of the Distributed Random Forest that utilizes randomized split thresholds to further minimize variance. Crucially, the XRT algorithm was automatically selected by the pipeline specifically because it outperformed the benchmark GLM in predictive capacity during internal cross‐validation. To maximize predictive accuracy and systematically prevent overfitting, the algorithm employed an automated early stopping mechanism. Specifically, the model dynamically extended the number of trees and halted the training process when the validation error (logarithmic loss) failed to yield relative improvement over consecutive scoring intervals, thereby determining the optimal tree architecture automatically without requiring manual user‐defined parameter boundaries. Model performance was definitively established based on the Area Under the Receiver Operating Characteristic Curve (AUC) on the 35% internal validation set.

#### 2.5.3 Model Interpretability via SHAP

To overcome the inherent “black‐box” limitations of complex machine learning models and to provide the rigorous clinical interpretability typically associated with simple additive models, SHapley Additive exPlanations (SHAP) theory was incorporated. By mathematical definition, SHAP is an additive feature attribution method. SHAP values were calculated to estimate the marginal additive contribution of each micro‐arousal and hypoxemic feature. SHAP summary plots were generated to rank global feature importance and illustrate the directionality of variable impacts across the cohort, while SHAP waterfall plots were utilized to delineate the specific, non‐linear predictive thresholds and the linear addition of feature impacts for individual observations.

All traditional statistical procedures and machine learning pipelines were executed utilizing R software (version 4.1.3). The XRT algorithm and automated model optimization were specifically implemented via the h2o package. A two‐tailed *p* value of < 0.05 was considered to indicate statistical significance for all conventional inferential tests.

## 3 Results

### 3.1 Baseline Characteristics

A total of 967 patients were included in this study. The average age was 48.27 ± 13.23 years. Most participants were male and exhibited moderate to severe sleep apnea, with a mean AHI of 46.87 ± 25.42. The mean minimum nocturnal oxygen saturation was 75.63% ± 12.90%, and the mean arousal index was 27.80 ± 20.14 events per hour. Memory decline was commonly reported among participants. Comorbidities were frequent, with hypertension present in 44.0% of the cohort, diabetes in 17.2%, and heart disease in 17.4%. The majority of patients were overweight or obese, with a mean BMI of 27.21 ± 4.27 kg/m^2^. Further details on sleep architecture, respiratory events, and additional clinical variables are provided in Table 1.

**TABLE 1:** Baseline characteristics of the study population.

Variables	$\bar{x}\pm s/N\left(\%\right)$
Age, years	48.27 ± 13.23
AHI index	46.87 ± 25.42
Minimum SpO_2_ (%)	75.63 ± 12.90
Sleep latency (min)	8.83 ± 16.13
REM duration (min)	56.64 ± 28.01
N1 duration (min)	177.32 ± 87.57
N2 duration (min)	160.03 ± 75.50
N3 duration (min)	26.33 ± 29.81
REM arousals	20.92 ± 20.29
NREM arousals	132.79 ± 115.65
Total arousals	170.63 ± 133.65
Respiratory event‐related REM arousals	1.09 ± 2.03
Respiratory event‐related NREM arousals	8.13 ± 14.91
Total respiratory event‐related arousals	9.96 ± 16.50
Spontaneous REM arousals	8.43 ± 12.28
Spontaneous NREM arousals	49.04 ± 55.05
Total spontaneous arousals	42.06 ± 27.84
Arousal index (events/h)	27.80 ± 20.14
AHI index	46.87 ± 25.42
Longest obstructive event (s)	53.85 ± 25.91
Obstructive event‐related desaturation count	195.87 ± 210.78
Longest obstructive desaturation (s)	53.21 ± 26.25
Hypopnea count (n)	117.44 ± 89.02
Longest hypopnea (s)	51.95 ± 16.14
Hypopnea‐related desaturation count (n)	84.32 ± 76.36
Longest hypopnea desaturation (s)	50.78 ± 15.97
Minutes with SpO_2_ < 80%	0.80 ± 3.06
Minutes with SpO_2_ < 85%	1.77 ± 5.42
Minutes with SpO_2_ < 90%	4.93 ± 9.92
Minutes with SpO_2_ < 95%	31.77 ± 37.81
NREM oxygen desaturation index	39.27 ± 29.06
Mean SpO_2_ (%)	93.60 ± 2.84
Minimum SpO_2_ (%)	75.63 ± 12.90
AHI in REM	45.75 ± 20.25
AHI in NREM	43.96 ± 26.08
AHI total	46.87 ± 25.42
RDI in REM	45.75 ± 20.25
RDI in NREM	43.96 ± 26.08
RDI total	46.87 ± 25.42
Nocturnal awakening	449 (50.7)
Epworth Sleepiness Scale	6.54 ± 5.42
Irritability compared to before	420 (47.5)
Hypertension	389 (44.0)
Heart disease	154 (17.4)
Diabetes	152 (17.2)
Cerebrovascular disease	119 (13.5)
Thyroid disease	113 (12.8)
GERD	304 (34.4)
Chronic rhinitis	319 (36.1)
Pharyngitis	340 (38.5)
History of upper airway surgery	100 (11.3)
Alcohol use	346 (39.2)
Smoking	333 (37.7)
Parental snoring	563 (64.1)
BMI (kg/m^2^)	27.21 ± 4.27
Neck circumference (cm)	39.85 ± 4.83
Waist circumference (cm)	96.38 ± 10.65
Bedtime systolic BP (mmHg)	134.11 ± 17.95
Bedtime diastolic BP (mmHg)	88.73 ± 12.68
Morning systolic BP (mmHg)	136.99 ± 18.09
Morning diastolic BP (mmHg)	93.94 ± 13.02
Pharyngeal stenosis	
None	130 (14.9)
Mild	251 (28.9)
Mild–Moderate	213 (24.5)
Severe	276 (31.7)
Inferior turbinate hypertrophy	423 (48.6)
Nasal septum deviation	313 (36.0)

### 3.2 Comparison of Characteristics Among Groups With Different Degrees of Memory Decline

#### 3.2.1 Comparison of Characteristics Between Memory‐Normal and Memory‐Decline Groups

Compared with the memory‐normal group, the memory‐decline group was older and showed higher prevalences of hypertension, heart disease, diabetes, cerebrovascular disease, and thyroid disease (all *p* ≤ 0.035). Upper‐airway conditions were more frequent in the memory‐decline group—reflux esophagitis, chronic rhinitis, pharyngitis, and prior upper‐airway surgery (all *p* ≤ 0.001)—with nasal septum deviation higher (*p* = 0.015) and inferior turbinate hypertrophy similar. Anthropometry and hemodynamics showed smaller neck circumference and lower pre‐sleep and morning diastolic blood pressure in the memory‐decline group (*p* = 0.003, 0.036, 0.010), while waist circumference and systolic blood pressure did not differ. Oropharyngeal stenosis distributions differed overall (*p* = 0.017), driven by a higher Level‐1 proportion in the memory‐decline group. Alcohol use, smoking, and parental snoring histories were comparable, Table 2.

**TABLE 2:** Comparison of characteristics memory‐normal and memory‐decline groups.

Variables	Memory‐normal (*N* = 408)	Memory‐decline (*N* = 476)	$t/x^{2}$	*p*
Age (years)	45.95 ± 12.47	50.24 ± 13.51	23.745	< 0.001
Hypertension	164 (40.2)	225 (47.3)	4.460	0.035
Heart disease	46 (11.3)	108 (22.7)	19.897	< 0.001
Diabetes	36 (8.8)	116 (24.4)	37.292	< 0.001
Cerebrovascular disease	25 (6.1)	94 (19.7)	34.986	< 0.001
Thyroid disease	21 (5.1)	92 (19.3)	39.626	< 0.001
Reflux esophagitis	111 (27.2)	193 (40.5)	17.328	< 0.001
Chronic rhinitis	123 (30.1)	196 (41.2)	11.587	0.001
Pharyngitis	129 (31.6)	211 (44.3)	14.995	< 0.001
History of upper airway surgery	22 (5.4)	78 (16.4)	26.470	< 0.001
Alcohol history	150 (36.9)	196 (41.2)	1.719	0.190
Smoking history	140 (34.4)	193 (40.5)	3.530	0.060
Parental snoring history	271 (66.9)	292 (61.7)	2.544	0.111
BMI (kg/m^2^)	27.28 ± 4.69	26.92 ± 4.52	0.817	0.366
Neck circumference (cm)	40.37 ± 4.25	39.35 ± 5.29	8.925	0.003
Waist circumference (cm)	96.90 ± 10.07	95.87 ± 11.21	1.668	0.197
Pre‐sleep systolic BP (mmHg)	134.91 ± 17.24	133.35 ± 18.64	1.348	0.246
Pre‐sleep diastolic BP (mmHg)	89.74 ± 12.80	87.77 ± 12.49	4.393	0.036
Morning systolic BP (mmHg)	137.36 ± 19.19	136.62 ± 16.97	0.252	0.616
Morning diastolic BP (mmHg)	95.33 ± 13.12	92.59 ± 12.80	6.657	0.010
Inferior turbinate hypertrophy	196 (48.9)	227 (48.4)	0.019	0.888
Nasal septum deviation	127 (31.8)	186 (39.7)	5.859	0.015
Oropharyngeal stenosis			10.222	0.017
I	67 (16.7)	63 (13.4)		
II	95 (23.7)	156 (33.3)		
III	107 (26.7)	106 (22.6)		
IV	132 (32.9)	144 (30.7)		

#### 3.2.2 Comparison of Polysomnographic Parameters Memory‐Normal and Memory‐Decline Groups

The memory‐decline group was characterized by a hypopnea‐predominant, mild‐hypoxemia phenotype. The cumulative event burden, as quantified by the AHI, was comparable, but desaturation morphology differed: the memory‐decline group had shorter maximal obstructive events, fewer and shorter desaturation‐accompanied obstructive events, and a lower NREM oxygen desaturation index. In contrast, hypopnea counts were higher and awake time with SpO_2_ < 95% was longer, while lowest nocturnal saturation was paradoxically higher. This profile indicates shallower but more persistent oxygen debt rather than deep, brief nadirs. Details of these comparisons are presented in Table 3.

**TABLE 3:** Comparison of polysomnographic parameters memory‐normal and memory‐decline groups.

Variables	Memory‐normal (*N* = 408)	Memory‐decline (*N* = 476)	$t/x^{2}$	*p*
AHI index	48.64 ± 25.61	45.82 ± 25.07	2.729	0.099
Nadir SpO_2_ (%)	74.39 ± 14.42	76.68 ± 11.42	6.930	0.009
Sleep latency	9.06 ± 16.84	8.47 ± 15.47	0.292	0.589
R duration	57.58 ± 27.52	56.12 ± 28.45	0.601	0.438
N1 duration	181.20 ± 89.35	175.14 ± 86.85	1.041	0.308
N2 duration	158.53 ± 74.71	161.56 ± 76.80	0.351	0.554
N3 duration	26.65 ± 30.60	25.78 ± 29.33	0.188	0.665
REM arousals	21.96 ± 20.80	20.05 ± 19.99	1.924	0.166
NREM arousals	138.46 ± 121.85	128.42 ± 110.97	1.641	0.201
Total arousals	178.34 ± 140.13	165.18 ± 129.13	2.109	0.147
Breathing event REM arousals	1.01 ± 1.82	1.19 ± 2.27	1.599	0.206
Breathing event NREM arousals	8.05 ± 13.29	8.51 ± 16.85	0.201	0.653
Total breathing event arousals	9.79 ± 14.95	10.46 ± 18.52	0.343	0.558
Spontaneous REM arousals	7.91 ± 11.31	8.35 ± 12.81	0.281	0.596
Spontaneous NREM arousals	44.62 ± 47.80	50.08 ± 57.95	2.285	0.131
Total arousal number	43.89 ± 30.18	41.68 ± 26.84	1.329	0.249
Arousal index	28.85 ± 20.49	26.98 ± 20.06	1.867	0.172
AHI index	48.64 ± 25.61	45.82 ± 25.07	2.729	0.099
Longest obstructive event	57.49 ± 27.93	51.42 ± 23.36	12.357	< 0.001
Total obstructive with desaturation	219.70 ± 222.13	180.33 ± 200.03	7.685	0.006
Longest obstructive with desaturation	57.08 ± 28.13	50.72 ± 23.77	13.263	< 0.001
Total hypopnea	110.40 ± 81.91	122.25 ± 93.39	3.960	0.047
Longest hypopnea	51.58 ± 17.07	52.55 ± 15.77	0.764	0.382
Total hypopnea with desaturation	81.10 ± 70.02	87.00 ± 81.22	1.315	0.252
Longest hypopnea with desaturation	50.47 ± 16.80	51.31 ± 15.60	0.595	0.441
WK O2 < 80 min	0.89 ± 3.16	0.77 ± 3.18	0.311	0.577
WK O2 < 85 min	1.93 ± 5.32	1.66 ± 5.70	0.522	0.470
WK O2 < 90 min	5.01 ± 9.02	4.86 ± 10.78	0.047	0.827
WK O2 < 95 min	27.71 ± 31.18	33.71 ± 40.77	5.884	0.015
NREM oxygen desaturation index	41.77 ± 29.54	37.72 ± 28.51	4.290	0.039
TOTAL mean O2	93.54 ± 3.08	93.77 ± 2.62	1.489	0.223
Lowest O2 sat	74.39 ± 14.42	76.68 ± 11.42	6.930	0.009
AHIREM	45.27 ± 20.29	46.16 ± 19.73	0.432	0.511
AHINREM	45.86 ± 26.34	42.68 ± 25.60	3.300	0.070
AHITST	48.64 ± 25.61	45.82 ± 25.07	2.729	0.099
RDIREM	45.27 ± 20.29	46.16 ± 19.73	0.432	0.511
RDINREM	45.86 ± 26.34	42.68 ± 25.60	3.300	0.070
RDITST	48.64 ± 25.61	45.82 ± 25.07	2.729	0.099

#### 3.2.3 Association Between Nocturnal Hypoxemia and Sleep Fragmentation

A comprehensive correlation analysis was conducted among multiple micro‐arousal and oxygen desaturation variables (Table 4). The following findings were observed: There are strong positive correlations between total REM micro‐arousals, total NREM micro‐arousals, and total micro‐arousals, indicating substantial overlap or shared features among these events. Several variables involving respiratory event‐related arousals are also moderately to strongly correlated with overall arousal and oxygen desaturation indices. Most correlations with oxygen saturation and hypopnea indices are moderate and in the expected directions (e.g., more arousals associated with lower oxygen levels).

**TABLE 4:** Association between nocturnal hypoxemia and sleep fragmentation.

Row	Column	*r*	*p*
Tot_REM_MA	Tot_NREM_MA	0.607	< 0.001
Tot_REM_MA	Tot_MA	0.679	< 0.001
Tot_NREM_MA	Tot_MA	0.991	< 0.001
Tot_REM_MA	REM_MA_RE	0.387	< 0.001
Tot_NREM_MA	REM_MA_RE	0.185	< 0.001
Tot_MA	REM_MA_RE	0.211	< 0.001
Tot_REM_MA	NREM_MA_RE	0.183	< 0.001
Tot_NREM_MA	NREM_MA_RE	0.412	< 0.001
Tot_MA	NREM_MA_RE	0.394	< 0.001
REM_MA_RE	NREM_MA_RE	0.397	< 0.001
Tot_REM_MA	Tot_MA_RE	0.211	< 0.001
Tot_NREM_MA	Tot_MA_RE	0.404	< 0.001
Tot_MA	Tot_MA_RE	0.393	< 0.001
REM_MA_RE	Tot_MA_RE	0.494	< 0.001
NREM_MA_RE	Tot_MA_RE	0.991	< 0.001
Tot_REM_MA	Spon_REM_MA	0.691	< 0.001
Tot_NREM_MA	Spon_REM_MA	0.308	< 0.001
Tot_MA	Spon_REM_MA	0.362	< 0.001
REM_MA_RE	Spon_REM_MA	0.418	< 0.001
NREM_MA_RE	Spon_REM_MA	0.12	< 0.001
Tot_MA_RE	Spon_REM_MA	0.159	< 0.001
Tot_REM_MA	Spon_NREM_MA	0.459	< 0.001
Tot_NREM_MA	Spon_NREM_MA	0.506	< 0.001
Tot_MA	Spon_NREM_MA	0.512	< 0.001
REM_MA_RE	Spon_NREM_MA	0.309	< 0.001
NREM_MA_RE	Spon_NREM_MA	0.217	< 0.001
Tot_MA_RE	Spon_NREM_MA	0.24	< 0.001
Spon_REM_MA	Spon_NREM_MA	0.731	< 0.001
Tot_REM_MA	Tot_MA_all	−0.101	0.002
Tot_NREM_MA	Tot_MA_all	0.154	< 0.001
Tot_MA	Tot_MA_all	0.196	< 0.001
REM_MA_RE	Tot_MA_all	−0.162	< 0.001
NREM_MA_RE	Tot_MA_all	0.014	0.662
Tot_MA_RE	Tot_MA_all	0.013	0.692
Spon_REM_MA	Tot_MA_all	−0.173	< 0.001
Spon_NREM_MA	Tot_MA_all	−0.100	0.002
Tot_REM_MA	MA_index	0.582	< 0.001
Tot_NREM_MA	MA_index	0.928	< 0.001
Tot_MA	MA_index	0.936	< 0.001
REM_MA_RE	MA_index	0.150	< 0.001
NREM_MA_RE	MA_index	0.373	< 0.001
Tot_MA_RE	MA_index	0.369	< 0.001
Spon_REM_MA	MA_index	0.324	< 0.001
Spon_NREM_MA	MA_index	0.502	< 0.001
Tot_MA_all	MA_index	0.319	< 0.001
Tot_REM_MA	Tot_OBD	0.344	< 0.001
Tot_NREM_MA	Tot_OBD	0.601	< 0.001
Tot_MA	Tot_OBD	0.605	< 0.001
REM_MA_RE	Tot_OBD	−0.149	< 0.001
NREM_MA_RE	Tot_OBD	0.029	0.368
Tot_MA_RE	Tot_OBD	0.001	0.976
Spon_REM_MA	Tot_OBD	−0.126	< 0.001
Spon_NREM_MA	Tot_OBD	−0.099	0.002
Tot_MA_all	Tot_OBD	0.338	< 0.001
MA_index	Tot_OBD	0.569	< 0.001
Tot_REM_MA	Long_OBD	0.21	< 0.001
Tot_NREM_MA	Long_OBD	0.332	< 0.001
Tot_MA	Long_OBD	0.341	< 0.001
REM_MA_RE	Long_OBD	−0.146	< 0.001
NREM_MA_RE	Long_OBD	−0.018	0.57
Tot_MA_RE	Long_OBD	−0.042	0.188
Spon_REM_MA	Long_OBD	−0.072	0.026
Spon_NREM_MA	Long_OBD	−0.075	0.019
Tot_MA_all	Long_OBD	0.264	< 0.001
MA_index	Long_OBD	0.376	< 0.001
Tot_OBD	Long_OBD	0.626	< 0.001
Tot_REM_MA	Tot_HYPO	−0.071	0.027
Tot_NREM_MA	Tot_HYPO	−0.204	< 0.001
Tot_MA	Tot_HYPO	−0.203	< 0.001
REM_MA_RE	Tot_HYPO	0.085	0.008
NREM_MA_RE	Tot_HYPO	0.002	0.949
Tot_MA_RE	Tot_HYPO	0.015	0.651
Spon_REM_MA	Tot_HYPO	0.069	0.032
Spon_NREM_MA	Tot_HYPO	0.043	0.178
Tot_MA_all	Tot_HYPO	−0.206	< 0.001
MA_index	Tot_HYPO	−0.264	< 0.001
Tot_OBD	Tot_HYPO	−0.447	< 0.001
Long_OBD	Tot_HYPO	−0.459	< 0.001
Tot_REM_MA	Long_HYPO	−0.148	< 0.001
Tot_NREM_MA	Long_HYPO	−0.252	< 0.001
Tot_MA	Long_HYPO	−0.254	< 0.001
REM_MA_RE	Long_HYPO	0.043	0.178
NREM_MA_RE	Long_HYPO	−0.06	0.063
Tot_MA_RE	Long_HYPO	−0.048	0.133
Spon_REM_MA	Long_HYPO	0.032	0.327
Spon_NREM_MA	Long_HYPO	0.033	0.299
Tot_MA_all	Long_HYPO	−0.17	< 0.001
MA_index	Long_HYPO	−0.285	< 0.001
Tot_OBD	Long_HYPO	−0.432	< 0.001
Long_OBD	Long_HYPO	−0.193	< 0.001
Tot_HYPO	Long_HYPO	0.397	< 0.001
Tot_REM_MA	HYPO_DESAT	−0.057	0.079
Tot_NREM_MA	HYPO_DESAT	−0.141	< 0.001
Tot_MA	HYPO_DESAT	−0.140	< 0.001
REM_MA_RE	HYPO_DESAT	−0.073	0.024
NREM_MA_RE	HYPO_DESAT	−0.105	0.001
Tot_MA_RE	HYPO_DESAT	−0.110	0.001
Spon_REM_MA	HYPO_DESAT	−0.009	0.769
Spon_NREM_MA	HYPO_DESAT	−0.028	0.385
Tot_MA_all	HYPO_DESAT	−0.134	< 0.001
MA_index	HYPO_DESAT	−0.199	< 0.001
Tot_OBD	HYPO_DESAT	−0.266	< 0.001
Long_OBD	HYPO_DESAT	−0.317	< 0.001
Tot_HYPO	HYPO_DESAT	0.919	< 0.001
Long_HYPO	HYPO_DESAT	0.367	< 0.001
Tot_REM_MA	Long_HYPO_DESAT	−0.145	< 0.001
Tot_NREM_MA	Long_HYPO_DESAT	−0.251	< 0.001
Tot_MA	Long_HYPO_DESAT	−0.251	< 0.001
REM_MA_RE	Long_HYPO_DESAT	0.027	0.402
NREM_MA_RE	Long_HYPO_DESAT	−0.080	0.012
Tot_MA_RE	Long_HYPO_DESAT	−0.070	0.029
Spon_REM_MA	Long_HYPO_DESAT	0.023	0.468
Spon_NREM_MA	Long_HYPO_DESAT	0.018	0.585
Tot_MA_all	Long_HYPO_DESAT	−0.155	< 0.001
MA_index	Long_HYPO_DESAT	−0.282	< 0.001
Tot_OBD	Long_HYPO_DESAT	−0.409	< 0.001
Long_OBD	Long_HYPO_DESAT	−0.184	< 0.001
Tot_HYPO	Long_HYPO_DESAT	0.383	< 0.001
Long_HYPO	Long_HYPO_DESAT	0.976	< 0.001
HYPO_DESAT	Long_HYPO_DESAT	0.374	< 0.001

### 3.3 Interpretable Machine Learning for Sleep Fragmentation

#### 3.3.1 Comparison of Characteristics Between the Training and Validation Datasets

To further elucidate the clinical implications of sleep fragmentation, the cumulative micro‐arousal burden was operationalized as a binary categorical outcome. Participants were stratified into a high micro‐arousal burden group and a low micro‐arousal burden group utilizing the median total micro‐arousal count of the analytical sample as a predefined objective threshold (median = 137 events). Specifically, individuals demonstrating ≥ 137 total micro‐arousal events during the polysomnographic recording were designated to the high‐burden phenotype (coded as 1), whereas those with < 137 events were assigned to the low‐burden phenotype (coded as 0).

The total analytic sample was randomly partitioned into a training dataset (*N* = 629) and an internal validation dataset (*N* = 338). As detailed in Table 5, there were no statistically significant differences between the training and validation sets across any of the assessed polysomnographic, respiratory, or nocturnal hypoxemia parameters (all *p* > 0.05). Both datasets exhibited highly comparable burdens of obstructive and hypopneic events, with no significant divergence in total event counts, maximum duration, or desaturation‐associated episodes. Similarly, indices of nocturnal hypoxic severity, including cumulative exposure below critical clinical thresholds (e.g., time with SpO_2_ < 90%, *p* = 0.610), NREM oxygen desaturation index (*p* = 0.942), and nadir oxygen saturation (75.60% ± 13.20% vs. 75.68% ± 12.34%, *p* = 0.925)—demonstrated robust consistency between the two datasets. Collectively, these findings validate integrity of the data partitioning process, ensuring that subsequent machine learning models were derived and validated on unbiased, structurally identical datasets, Table 5.

**TABLE 5:** Comparison of hypoxemia‐related parameters between validation data and train groups.

Characteristic	Validation data (*N* = 338)	Train data (*N* = 629)	*t*	*p*
Total obstructive events	199.94 ± 212.86	193.68 ± 209.80	0.441	0.660
Longest obstructive	53.39 ± 25.33	53.12 ± 26.75	0.153	0.879
Total hypopnea	117.80 ± 91.45	117.24 ± 87.76	0.092	0.927
Longest hypopnea	52.39 ± 16.02	51.71 ± 16.21	0.628	0.530
Total hypopnea	85.09 ± 78.24	83.91 ± 75.39	0.230	0.818
Longest hypopnea	50.97 ± 15.64	50.67 ± 16.15	0.270	0.787
WK O2 < 80 min	0.89 ± 3.59	0.75 ± 2.74	0.654	0.513
WK O2 < 85 min	1.99 ± 6.57	1.66 ± 4.69	0.900	0.368
WK O2 < 90 min	5.15 ± 11.53	4.81 ± 8.93	0.510	0.610
WK O2 < 95 min	31.52 ± 34.24	31.91 ± 39.62	−0.154	0.878
NREM oxygen desaturation index	39.18 ± 28.67	39.32 ± 29.29	−0.073	0.942
TOTAL mean O2	93.58 ± 2.72	93.61 ± 2.91	−0.192	0.848
Nadir SpO_2_ (%)	75.68 ± 12.34	75.60 ± 13.20	0.095	0.925

#### 3.3.2 Model Building for Interpretable Machine Learning

**FIGURE 1:**
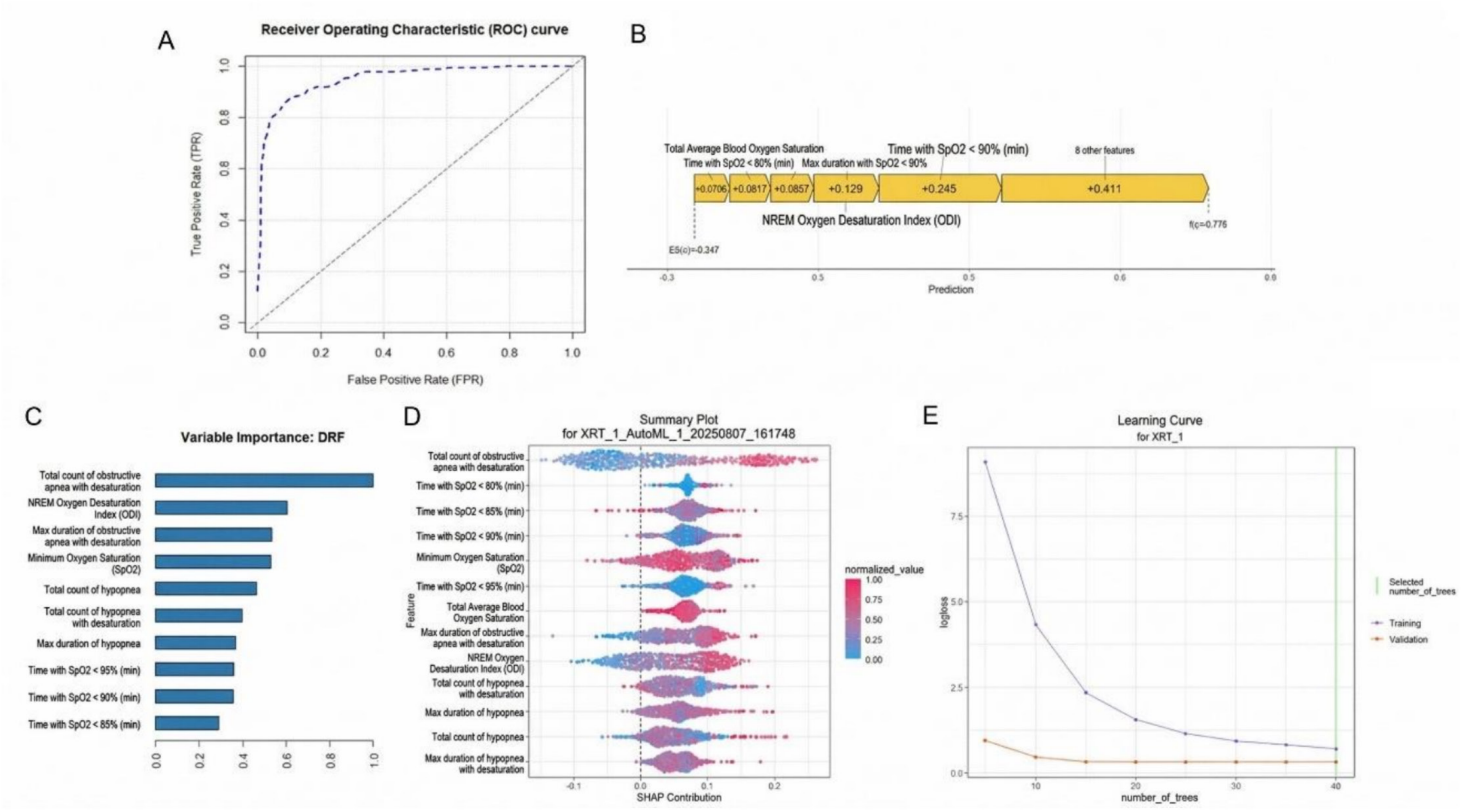
Interpretability of machine learning model for predicting total micro‐arousal events: variable importance, SHAP contributions, and model performance. (A) Receiver Operating Characteristic (ROC) curve for the prediction model. (B) SHAP waterfall plot showing feature contributions to a single prediction. (C) Variable importance plot based on the Distributed Random Forest (DRF) model. (D) SHAP summary plot demonstrating the impact of features across the dataset. (E) Learning curve depicting model performance with increasing number of trees.

The interpretability analysis of the machine learning model predicting total micro‐arousal events revealed robust predictive performance, as demonstrated by the ROC curve (Panel A) with high true positive rates across a range of false positive rates. Feature importance analysis using the Distributed Random Forest algorithm (Panel C) identified the total number of obstructive desaturation events, NREM oxygen desaturation index, and the longest obstructive desaturation duration as the most influential predictors. The SHAP summary plot (Panel D) further confirmed that features related to obstructive and hypoventilation‐associated desaturation events, as well as time spent with low oxygen saturation, contributed substantially to the model output. The SHAP waterfall plot (Panel B) illustrated the cumulative contribution of individual features toward a single prediction, highlighting the additive effect of both obstructive and hypoventilation indices. The learning curve (Panel E) indicated that the model achieved stable validation loss with an increasing number of trees, suggesting minimal overfitting and good generalizability. Overall, the interpretability analyses emphasize the central role of nocturnal oxygen desaturation and ventilatory event metrics in predicting micro‐arousal events, providing a transparent and biologically plausible rationale for the model's predictions, Figure 1.

### 3.4 Interpretable Machine Learning for Memory Decline

#### 3.4.1 Comparison of Characteristics Between the Training and Validation Datasets

There were no statistically significant differences between the training and validation sets across any of the assessed sleep fragmentation and micro‐arousal parameters (all *p* > 0.05). Both datasets exhibited highly comparable stage‐specific arousal burdens, with no significant divergence in total, REM‐specific, or NREM‐specific micro‐arousal counts. Similarly, indices of arousal etiology and overall fragmentation severity, including spontaneous NREM arousals (*p* = 0.062), total breathing event‐related arousals (*p* = 0.561), and the overarching arousal index (27.36 ± 19.88 events/h vs. 28.73 ± 20.98 events/h, *p* = 0.346), demonstrated robust consistency between the two datasets. Collectively, these findings validate the integrity of the data partitioning process, ensuring that subsequent machine learning models were derived and validated on unbiased, structurally identical datasets in Table 6.

**TABLE 6:** Comparison of arousal parameters between validation data and train groups.

Variables	Validation data (*N* = 309)	Train data (*N* = 575)	*t*	*p*
REM arousals	22.28 ± 21.28	20.21 ± 19.86	1.412	0.158
NREM arousals	141.18 ± 120.97	128.69 ± 113.36	1.496	0.135
Total arousals	180.65 ± 140.85	166.20 ± 130.66	1.491	0.137
Breathing event REM arousals	1.23 ± 2.27	1.04 ± 1.96	1.265	0.207
Breathing event NREM arousals	8.58 ± 16.41	8.14 ± 14.69	0.390	0.697
Total breathing event arousals	10.62 ± 18.36	9.90 ± 16.16	0.581	0.561
Spontaneous REM arousals	8.77 ± 13.20	7.81 ± 11.53	1.073	0.284
Spontaneous NREM arousals	52.15 ± 59.72	45.09 ± 49.79	1.872	0.062
Total arousal number	41.99 ± 28.94	43.09 ± 28.19	−0.540	0.590
Arousal index	28.73 ± 20.98	27.36 ± 19.88	0.944	0.346

**FIGURE 2:**
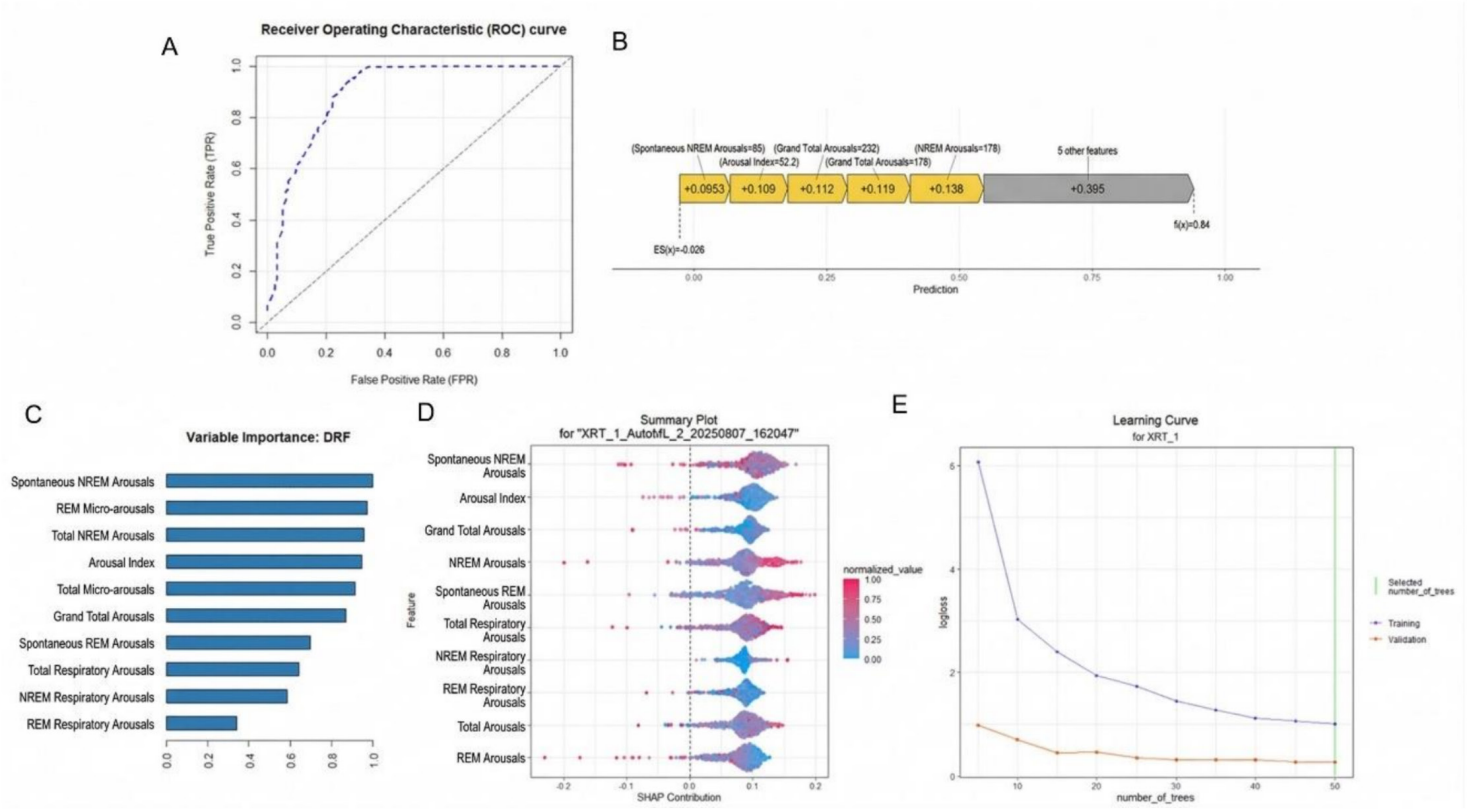
Interpretability of machine learning model for predicting memory decline: variable importance, SHAP contributions, and model performance (A) Receiver Operating Characteristic (ROC) curve for the memory decline prediction model. (B) SHAP waterfall plot illustrating the contribution of each micro‐arousal‐related feature to a single prediction. (C) Variable importance plot based on the Distributed Random Forest (DRF) model, ranking micro‐arousal features. (D) SHAP summary plot displaying the impact and directionality of individual micro‐arousal variables across the population. (E) Learning curve showing model performance (logloss) as a function of the number of trees.

The interpretability analysis of the machine learning model for predicting memory decline highlighted strong predictive performance, as shown by the ROC curve (Panel A), indicating a high discriminative capacity. According to the variable importance plot (Panel C), the most influential features were the total number of spontaneous NREM micro‐arousals, total REM micro‐arousals, and the overall arousal index, with respiratory event‐related arousal counts also contributing substantially. The SHAP summary plot (Panel D) confirmed that both spontaneous and event‐related micro‐arousals—particularly those occurring during NREM and REM sleep—had a significant impact on model predictions for memory decline. The SHAP waterfall plot (Panel B) detailed how cumulative effects of multiple micro‐arousal variables and indices combined to shift individual predictions. Finally, the learning curve (Panel E) demonstrated stable validation loss and minimal overfitting with increasing model complexity. Collectively, these interpretability analyses provide transparent evidence that micro‐arousal dynamics, especially their frequency and association with respiratory events, play a key role in predicting memory decline, Figure 2.

## 4 Discussion

Our analysis delineates a critical phenotypic divergence in the nocturnal hypoxemia morphology, prioritizing the dynamic architecture of oxygen desaturation over traditional cumulative event frequencies. While global severity markers (e.g., AHI) remained comparable between the cohorts, the memory‐decline group exhibited a distinct hypopnea‐dominant pattern. This profile was characterized by prolonged exposure to mild hypoxemia, whereas extreme obstructive events yielding deep nadirs were paradoxically less prominent. Furthermore, the overall micro‐arousal burden did not differ significantly. This distinct pattern strongly suggests a potential dose‐dependent association between continuous, low‐grade hypoxic exposure, and cognitive vulnerability, indicating that true biological impact diverges from strictly event‐count‐based metrics. This pattern suggests a potential dose‐dependent association between hypoxemic burden and cognitive variance, rather than a strictly event‐count‐based relationship. Prior cohorts align with this interpretation. In older women, sleep‐disordered breathing predicted incident MCI/dementia, and hypoxemia measures—not sleep fragmentation—tracked the risk signal. In older men, nocturnal hypoxemia associated with subsequent global cognitive decline. Community data from HypnoLaus also link lower mean nocturnal SpO_2_ and time below saturation thresholds with multi‐domain decline [19].

From a pathophysiological perspective, cumulative hypoxemia exposure—the time × depth area—appears to be more closely associated with memory vulnerability than rare deep nadirs. REM‐specific hypoxemia has been tied to memory impairment with CA1 hippocampal volume loss as a mediator, consistent with selective hippocampal vulnerability to sustained mild oxygen debt [20]. Standard event counts have inherent limitations. The AHI mixes apneas and hypopneas and fundamentally ignores the area of oxygen desaturation [21, 22]. Recent literature emphasizes the sleep‐apnea‐specific hypoxic burden (the sum of desaturation areas across events normalized by sleep time), which captures frequency, depth, and duration, often outperforming AHI for major clinical outcomes [22, 23]. A notable limitation of our current study is the absence of formal, integral‐based hypoxic burden quantification (i.e., area under the desaturation curve). This constraint restricts our ability to infer deep dose–response relationships. Nevertheless, our machine learning models successfully utilized standard threshold and duration metrics (e.g., T95, mean nocturnal SpO_2_) as robust predictors. Future cognitive models will benefit from incorporating hypoxic burden alongside conventional AHI to better represent true biological exposure.

Scoring choices shape phenotype detection. The 2012 AASM update and 2017 clarifications increased hypopnea capture and raised AHI in clinical populations, with population‐level effects on apparent severity and sex‐specific detection [24]. Prevalence and comorbidity associations vary under 3% desaturation/arousal versus 4% desaturation rules. Clear reporting of hypopnea criteria is required for comparability and to avoid misclassifying hypopnea‐dominant, light‐desaturation phenotypes. The longer awake time with SpO_2_ < 95% observed here suggests reduced gas‐exchange reserve or lung comorbidity that could amplify an all‐day light hypoxemia load. COPD literature links chronic hypoxemia to cognitive deficits and indicates potential benefit of correcting hypoxemia. Adjusting for lung disease markers or spirometry is advisable in sensitivity analyses [25]. Microarousal metrics did not emerge as prominent predictive features in our data, which mirrors prior observations that hypoxemia [26], rather than sleep fragmentation, better predicts incident cognitive impairment. Model exposures on two axes: frequency (AHI/RDI) and dose (T90/T95, mean nocturnal SpO_2_, hypoxic burden) [27].

Several limitations of this study warrant consideration. First, memory decline was evaluated utilizing a structured self‐reported questionnaire rather than objective neuropsychological testing (MoCA or MMSE). While this introduces potential susceptibility to recall bias and mood confounding—particularly given the high prevalence of daytime sleepiness and fatigue inherent to the OSA clinical phenotype—subjective memory complaints remain highly clinically relevant. Often conceptualized as SCD [28, 29, 30], these self‐perceptions function as highly sensitive early indicators of neurocognitive vulnerability that frequently precede objectively measurable deficits, directly reflecting patients' real‐world functional impairment and diminished quality of life [31, 32, 33]. Second, given the observational design, group comparisons remain susceptible to residual confounding. Furthermore, the observed effect sizes are relatively modest, and multiple testing may attenuate nominal statistical significance. Third, the lack of direct integral‐based hypoxic‐burden quantification limits the depth of dose–response inference. Consequently, future prospective longitudinal studies are warranted. Such studies should integrate comprehensive objective neuropsychological batteries, PROMs, and stage‐specific hypoxemia metrics alongside structural neuroimaging endpoints. This multidimensional approach is essential to unravel the exact temporal relationships and definitively test potential mediation pathways—such as hypoxic hippocampal injury—underlying both subjective and objective cognitive trajectories.

## 5 Conclusion

Memory decline aligns more with hypoxemia shape than with event counts. A hypopnea‐predominant phenotype, characterized by mild yet protracted nocturnal hypoxemia, provides a compelling pathophysiological framework and delineates actionable therapeutic targets for future prospective trials.

## Author Contributions

Xin Li: conceptualization, methodology, writing – original draft. Yingying Zhu and Fansu Meng: data curation, formal analysis. Kangan Lai and Yuling Liang: investigation, validation. Huang Ting: visualization, software. Zhengnan Mai: writing – review and editing. Liang Li: supervision, project administration, funding acquisition. All authors read and approved the final manuscript.

## Funding

This study was supported by the Zhongshan Social Public Welfare and Basic Research Project (grant no. 2021B1056).

## Ethics Statement

The study was approved by the institutional ethics committee of Zhongshan Hospital of Traditional Chinese Medicine (Approval No. 2021ZSZY‐LLK‐131). All participants provided informed consent.

## Conflicts of Interest

The authors declare no conflicts of interest.

## Data Availability

The data that support the findings of this study are available from the corresponding author upon reasonable request.
